# Acupuncture for management of lower urinary tract symptoms in Parkinson's disease: A protocol for the systematic review of randomized controlled trials

**DOI:** 10.1097/MD.0000000000009821

**Published:** 2018-02-09

**Authors:** Jong-In Kim, Tae-Young Choi, Ji Hee Jun, Hee Kang, Myeong Soo Lee

**Affiliations:** a Division of Acupuncture & Moxibustion Medicine, Kyung Hee Korean Medicine Hospital, Kyung Hee University, Seoul; b Clinical Research Division, Korea Institute of Oriental Medicine, Daejeon; c Graduate School of East-West Medical Science, Kyung Hee University, Yongin, Republic of Korea.

**Keywords:** acupuncture, lower urinary tract symptoms, Parkinson's disease, percutaneous tibial nerve stimulation, protocol, systematic review

## Abstract

**Background:**

Acupuncture is claimed to improve the lower urinary tract symptoms (LUTS). Currently, no systematic reviews are performed for acupuncture on LUTS in patients with Parkinson's diseases (PD). This review aims to evaluate the current evidence on the efficacy of acupuncture for the management of LUTS in PD.

**Methods and analyses:**

Eleven databases will be searched from their inception. These include PubMed, AMED, EMBASE, the Cochrane Library, 6 Korean medical databases, and 1 Chinese medical database. Study selection, data extraction, and assessment will be performed independently by 2 researchers. Risk of bias will be assessed with the Cochrane risk of bias assessment tool.

**Ethics and dissemination:**

Ethical approval will not be required, given that this protocol is for a systematic review. The systematic review will be published in a peer-reviewed journal and disseminated both electronically and in print. The review will be updated to inform and guide healthcare practice and policy.

Trial registration number: PROSPERO 2018 CRD42018083857

## 1 Introduction

### 1.1 Description of the condition

Lower urinary tract symptoms (LUTS) are among the most common types of associated autonomic dysfunctions in Parkinson's disease (PD).^[1–3]^ LUTS significantly influences the quality-of-life (QOL),^[4]^ early institutionalization, and health expenses in PD.^[5,6]^ Overactive bladder (OAB) symptom is the most common LUTS in PD patients.^[1]^

### 1.2 Description of the intervention

Acupuncture has been used to treat different conditions for at least 3000 years in East Asia. The acupuncture needle inserted into an acupuncture point produces de-qi sensation that causes the excitation of A-delta and C-fibers of the muscle tissue, resulting in afferent signals ascending to the brain via the dorsal horn cells of the spinal cord ^[7]^ Percutaneous tibial nerve stimulation (PTNS) is derived from acupuncture ^[8]^ and is the same as electroacupuncture procedure at ST36. PTNS involves a minimally invasive neuromodulation system delivering electrical stimulation to sacral nerve plexus through the percutaneous stimulation of the posterior tibial nerve plexus. One trial suggested that PTNS reduces LUTS in in PD patients.^[9]^

### 1.3 How the intervention might work

The mechanism of action of acupuncture for LUTS in PD is not precisely understood. Mechanical stimulation during acupuncture and/or PTNS seems to pass signals via the sensory ganglia to the spinal cord and via interneurons to modulate the activity of motor neurons in the brain stem network ^[10–12]^ that control autonomic function, including urogenital structures such as detrusor and sphincter muscles.^[13]^ However, reliable evidence is missing.

### 1.4 Why the intervention is important to this review

The complex pathophysiology of this bladder dysfunction makes LUTS unresponsive to levodopa, and the adverse events (AEs) on cognitive and gastrointestinal functions may limit the use of antimuscarinic drugs.^[14]^ Therefore, several options are for alleviatating storage LUTS ranging from behavioral modification,^[15]^ third-line surgical or invasive treatment options including deep brain stimulation,^[16]^ and intravesical botulinum toxin.^[17]^ In addition, the trials with these treatments suffered several caveats to elucidate placebo effect.^[14]^ Therefore, acupuncture may be 1 of options for managing LUTS in PD.

### 1.5 Objective

This review aims to systematically evaluate the evidence on the safety and effectiveness of acupuncture for treating LUTS in PD from randomized controlled trials (RCTs).

## 2 Methods

### 2.1 Study registration

This protocol has been registered on PROSPERO 2018 CRD 42018083857 (http://www.crd.york.ac.uk/PROSPERO/display_record.php?ID=CRD42018083857).

### 2.2 Criteria for considering studies for this review

#### 2.2.1 Types of studies

Prospective randomized controlled trials (RCTs) will be included. We will exclude observational, cohort, case–control, case series, qualitative studies, uncontrolled trials, and laboratory studies. No language restriction will be imposed.

#### 2.2.2 Types of participants

We will include patients with LUTS and PD regardless of age, sex, and race. We will only include studies in which an external set of criteria had been used to screen participants for the condition (e.g., criteria from the International Urogynecological Association/International Continence Society (ICS) joint report on the terminology for female pelvic floor dysfunction, the American Urological Association (AUA) or ICS).^[18–20]^

#### 2.2.3 Types of interventions and controls

Studies that have evaluated any type of invasive acupuncture with or without electrical stimulation will be included. PTNS, performed by urologists, will also be included because this procedure has originated from traditional acupuncture and follows the same method as that of electroacupuncture at SP6, on the medial aspect of the lower leg, 3 *cun* above the medial malleolus, on the posterior border of the medial aspect of the tibia.^[21]^ The treatment must involve needle insertion at acupuncture points, pain points, or trigger points and must be described as acupuncture. Studies investigating other methods of stimulating acupuncture points without needle insertion (acupressure, pressed studs, laser stimulation, etc.) will be included but analyzed separately. Control interventions may include treatments such as general conventional care (drugs, behavioral approach, etc.), sham treatment (interventions mimicking “true” acupuncture/true treatment but deviating in at least 1 aspect considered important by acupuncture theory, such as skin penetration or correct point location), or waiting list care. We will also include trials that have compared acupuncture plus another active treatment with same other active treatment alone. We will exclude RCTs in which 1 type of acupuncture is compared with a different type of acupuncture.

#### 2.2.4 Type of outcome measures

##### 2.2.4.1 Primary outcomes

(1) Urinary function: change in urgency, frequency of nocturia, and urgency incontinence.
(2) Total treatment efficacy: the number of patients whose LUTS have improved.
(3) Impact on symptoms measured by validated questionnaires, such as the Urinary Incontinence Questionnaire and Urogenital Distress Inventory,^[22]^ psychosocial adjustment to illness scale,^[23]^ Overactive Bladder Symptom Score, and the International Prostate Symptom Score.

##### 2.2.4.2 Secondary outcomes

(1) Quality of life: measured using a validated questionnaire, such as the ICIQ,^[24]^ Medical Outcomes Study Short-Form 20,^[25]^ and the King's Health Questionnaire.^[26]^
(2) Adverse events (AEs).

### 2.3 Search method for identifying the studies

#### 2.3.1 Electronic searches

Electronic databases searched from their inception will include MEDLINE, EMBASE, the Cochrane Central Register of Controlled Trials (CENTRAL), AMED, 6 Korean databases (KoreaMed, the Korean Traditional Knowledge Portal, Oriental Medicine Advanced Searching Integrated System (OASIS), DBpia, the Research Information Service System (RISS) and the Korean Studies Information Service System), and 1 Chinese database (China National Knowledge Infrastructure (CNKI)). Articles identified through reference lists of the included studies and relevant systematic reviews will be considered for inclusion based on their title. Study selection will be documented and summarized in a Preferred Reporting Items for Systematic Reviews and Meta-Analysis (PRISMA)-compliant flow chart (http://www.prisma-statement.org)

#### 2.3.2 Search of other resources

The authors will scan the reference lists and retrieve additional studies. In addition, authors will search the WHO International Clinical Trials Registry Platform (ICTRP) (http://apps.who.int/trialsearch/) and Google Scholar (http://scholar.google.co.kr/). Dissertations will be included. The ClinicalTrials.gov registry (http://clinicaltrials.gov/) will be searched for any unpublished trial.

#### 2.3.3 Search strategy

Our search strategy will include main keywords such as “acupuncture,” “PTNS,” “LUTS in Parkinson's disease,” and “OAB in Parkinson's disease” in English, Chinese, and Korean.

### 2.4 Data collection, extraction, and assessment

#### 2.4.1 Selection of studies

Two reviewers (JIK and TYC) will independently screen the titles and abstracts for searched studies and perform study selection and record their decisions according to predefined criteria. Another reviewer (MSL) will resolve disagreements in study selection. Study selection will be documented and summarized in a PRISMA flow diagram.

#### 2.4.2 Data extraction

All articles will be read by 2 independent reviewers (JIK and TYC) who will extract data from the articles according to predefined criteria. The extracted data will include the author name(s), year of publication, country, sample size, age and sex of the participants, acupuncture intervention, control intervention, main outcomes, and adverse effects. The extracted data will be tabulated (see online supplement 3) for future analysis. We will use the Grading of Recommendations Assessment, Development and Evaluation (GRADE) software to determine the quality of evidence based on the Cochrane Handbook for Systematic Reviews of Interventions to create a Summary of Findings table.^[27]^ Details regarding the acupuncture method and control interventions will be extracted based on revised Standards for Reporting Interventions in Clinical Trials of Acupuncture (STRICTA).^[28]^ When reported data are insufficient or unclear, an author will contact the first author or corresponding authors by e-mail or telephone to request missing or clarification data.

#### 2.4.3 Assessment of risk of bias

Quality assessment will be performed using the tool for “risk of bias” assessment from the Cochrane Handbook for Systematic Reviews of Interventions.^[29]^The following characteristics will be assessed: random sequence generation, allocation concealment, blinding of participants and personnel, blinding of outcome assessment, incomplete outcome data, selective outcome reporting, and other sources of bias (we will evaluate baseline imbalance). This review will use “L, U, and H” as the key for these evaluations, where “Low” (L) indicates a low risk of bias, “Unclear” (U) indicates that the risk of bias is uncertain, and “High” (H) indicates a high risk of bias. Disagreements will be resolved by discussion among all authors. Information regarding the risk of bias assessment for the included studies will be summarized in a table, and the results and implications will be critically discussed.

### 2.5 Data analysis

All statistical analyses will be conducted using the Cochrane Collaboration's software Review Manager (RevMan), v. 5.3 for Windows (The Nordic Cochrane Center, Copenhagen, Denmark). Differences between the intervention and control groups will be assessed. In the analysis of clinical efficacy, categorical data will be assessed in terms of risk ratios, and continuous data will be assessed in terms of mean difference (MD). Categorical and continuous variables will be expressed as efficacy values with 95% confidence intervals (CIs). In cases of outcome variables with different scales, standardized MD will be used instead of weighted MD. If we detect heterogeneity (defined by results of tests of heterogeneity that indicate *P* < 0.1 by chi-square test and Higgins *I*^2^≥50%), subgroup analyses will be performed to find the cause of clinical heterogeneity. A random effects model will be used to assess combined effect sizes from efficacy variables because substantial clinical heterogeneity is expected across the included studies based on the diversity of interventions, study design, and other conditions. Publication bias will be assessed using funnel plots and Egger's regression method.^[30]^ If missing data are detected, we will request any missing or incomplete information from the investigators of the original study.

Subgroup analysis will be conducted according to different control interventions (sham acupuncture vs conventional medication), the type of acupuncture (Chinese vs Western or PTNS), type of stimulation (manual vs electric), acupuncture points (1 or 2 points vs multiple points), and the design of the trial (acupuncture vs sham acupuncture; acupuncture vs conventional medication; acupuncture combined with conventional medication vs conventional medication). Where appropriate, sensitivity analysis will be performed to evaluate the robustness of the meta-analysis results. To verify consistency with other meta-analyses and meta-regression, the primary quality assessment will be a binary measure of allocation concealment.^[31]^

## 3 Discussion

Until now, no systematic review on the use of acupuncture for LUTS in PD has been published. The results of this systematic review evaluating the evidence on the safety and effectiveness of acupuncture for treating LUTS in PD from RCTs may be utilized by clinicians for managing LUTS in PD.

## 4 Contribution of authors

The protocol of a review was drafted by all authors. The search strategy was established by TYC, JHJ. Copies of studies will be obtained by JIK, TYC, and JHJ. Selection of the studies to include will be performed by JIK, TYC, and HK. MSL will act as an arbiter in the study selection stage. Extraction of data from studies will be conducted by TYC and JHJ. Entering data into RevMan 5.3.0 Version will be conducted by HK and JIK. Interpretation of results will be performed by all authors. The final review will be drafted and revised by all authors. The review will be updated by all authors.
